# The Impact of Glycemic Control on Retinal Photoreceptor Layers and Retinal Pigment Epithelium in Patients With Type 2 Diabetes Without Diabetic Retinopathy: A Follow-Up Study

**DOI:** 10.3389/fendo.2021.614161

**Published:** 2021-04-23

**Authors:** Fukashi Ishibashi, Aiko Kosaka, Mitra Tavakoli

**Affiliations:** ^1^ Internal Medicine, Ishibashi Clinic, Hiroshima, Japan; ^2^ Diabetes and Vascular Research Centre (DVRC), NIHR Exeter Clinical Research Facility, University of Exeter Medical School, Exeter, United Kingdom

**Keywords:** macular photoreceptor layers, retinal pigment epithelium, enface optical coherence tomography, glycemic control, type 2 diabetes, microvascular complications, diabetic neuropathy, diabetic retinopatathy

## Abstract

**Aims:**

To establish the sequential changes by glycemic control in the mean thickness, volume and reflectance of the macular photoreceptor layers (MPRLs) and retinal pigment epithelium in patients with type 2 diabetes without diabetic retinopathy.

**Methods:**

Thirty-one poorly controlled (HbA1c > 8.0%) patients with type 2 diabetes without diabetic retinopathy undergoing glycemic control and 39 control subjects with normal HbA1c levels (< 5.9%) underwent periodical full medical, neurological and ophthalmological examinations over 2 years. Glycemic variability was evaluated by standard deviation and coefficient of variation of monthly measured HbA1c levels and casual plasma glucose. 3D swept source-optical coherence tomography (OCT) and OCT-Explorer-generated enface thickness, volume and reflectance images for 9 subfields defined by Early Treatment Diabetic Retinopathy Study of 4 MPRLs {outer nuclear layer, ellipsoid zone, photoreceptor outer segment (PROS) and interdigitation zone} and retinal pigment epithelium were acquired every 3 months.

**Results:**

Glycemic control sequentially restored the thickness and volume at 6, 4 and 5 subfields of outer nuclear layer, ellipsoid zone and PROS, respectively. The thickness and volume of outer nuclear layer were restored related to the decrease in HbA1c and casual plasma glucose levels, but not related to glycemic variability and neurological tests. The reflectance of MPRLs and retinal pigment epithelium in patients was marginally weaker than controls, and further decreased at 6 or 15 months during glycemic control. The reduction at 6 months coincided with high HbA1c levels.

**Conclusion:**

Glycemic control sequentially restored the some MPRL thickness, especially of outer nuclear layer. In contrast, high glucose during glycemic control decreased reflectance and may lead to the development of diabetic retinopathy induced by glycemic control. The repeated OCT examinations can clarify the benefit and hazard of glycemic control to the diabetic retinopathy.

## Introduction

Diabetic retinopathy, the leading cause of blindness in working-age individuals has been viewed traditionally as a microvascular complication in diabetes. Indeed, the clinical classification system for diabetic retinopathy is based solely on the changes in the retinal microvasculature, because the microvasculature is visible using the routine fundus imaging. The advent of the high-resolution spectral-domain and swept-source-optical coherence tomography (OCT) and image segmentation algorithms permit depth-resolved enface OCT imaging for viewing the retinal layers in the coronal plane, enabling to measure the metrics (spatial thickness, volume and reflectance) of individual macular photoreceptor layers (MPRLs) and retinal pigment epithelium (RPE) (1).

It has been shown that glycemic variability induces retinal neurodegeneration in type 1 diabetes (2), but this has not been studied in type 2 diabetes.

However, studies on the impact of diabetes on the metrics of MPRLs and RPE using enface OCT had been cross-sectional (3) and inconsistent (4, 5). Because the thickness of individual MPRLs, except for outer nuclear layer (ONL), and RPE is < 20 µm, and because OCT image quality depends on the instrument reliability and measurement variability (6), the excellent OCT repeatability is essential to measure the metrics of the individual layers of MPRLs and RPE. We measured the metrics of RPE, because RPE has the close functional and morphological relationship with MPRLs.

For evaluating the impact of glycemic control on the structures of individual layers of MPRLs and RPE, the sequential measurements during follow-up are necessary. The sequential changes by glycemic control in the metrics of individual MPRLs and RPE using enface OCT in diabetes had never been studied.

The current study aimed to investigate the impact of glycemic control and glycemic variability on the sequential changes in the metrics of individual MPRLs and RPE in type 2 diabetic patients without diabetic retinopathy using enface OCT.

## Subjects And Methods

### Subjects

Thirty-one patients with type 2 diabetes under poor glycemic control (HbA1c > 8.0%) at the baseline undergoing a subsequent glycemic control, and 39 healthy gender- and age-matched control subjects with normal HbA1c levels (HbA1c < 5.9%) were studied. All subjects were enrolled at the period from January 2016 to December 2017 at the Ishibashi Clinic, Hiroshima Japan, and were followed up for 24 months. The exclusion criteria of all subjects were as follows; best-corrected visual acuity < 1.0, color blindness, diabetic retinopathy of any grade, macular edema, glaucoma, history of intraocular disease, refractive surgery, using hard contact lenses, neurodegenerative diseases, and significant media opacities. Written informed consent was obtained from all subjects based on the Declaration of Helsinki. The ethics committee of the Ishibashi Clinic approved the protocol of the present research.

### Clinical and Laboratory Data

The BMI, blood pressure, casual postprandial plasma glucose (CPPG), and HbA1c levels were measured monthly during the terms of study in patients with type 2 diabetes, and at the baseline and endpoint in control subjects. In patients, the standard deviation and coefficient of variation of CPPG and HbA1c levels over the whole follow-up period were calculated for estimating glycemic variability. The serum lipid levels (LDL-cholesterol, HDL-cholesterol and triglycerides), and urinary creatinine and albumin levels were assessed every 3 months in patients. An albumin-to-creatinine ratio > 30mg/g creatinine twice a year was labeled as nephropathy (7).

### Ophthalmic Examinations

The visual acuity was measured using the international type visual acuity chart (Tsutsumi, Tokyo, Japan). The color vision was assessed by the Ishihara color test. The bilateral fundus images were captured (the field of assessment: 45°) without pupil dilatation every 6 months for patients with type 2 diabetes, and at the baseline and endpoint for control subjects.

### Corneal Confocal Microscopy

All subjects were examined at the baseline and endpoint using a Heidelberg Retina Tomograph III *in vivo* corneal confocal microscope with Rostock Corneal Module (Heidelberg Engineering, Heidelberg, Germany) (8). Six high-quality images per subject from Bowman’s layer were captured for quantifying the following corneal nerve fiber (CNF) morphological parameters: 1) CNF density: total number of major nerve fibers/mm^2^ of corneal tissue; 2) CNF length: total length of all nerve fibers (mm/mm^2^); 3) corneal nerve branch density: number of branches emanating from all major nerve trunks/mm^2^; 4) beading frequency/0.1mm; and 5) bead size (μm^2^) (9). Except for bead size, all measurements were performed using ImageJ (Texelcraft, Tokyo, Japan). The examiners and team members analyzing the images were all blinded and masked to the study groups.

### 3D Swept-Source-OCT, Automated Segmentation, Mean Thickness, Volume, and Reflectance of MPRLs and RPE

**Figure 1:**
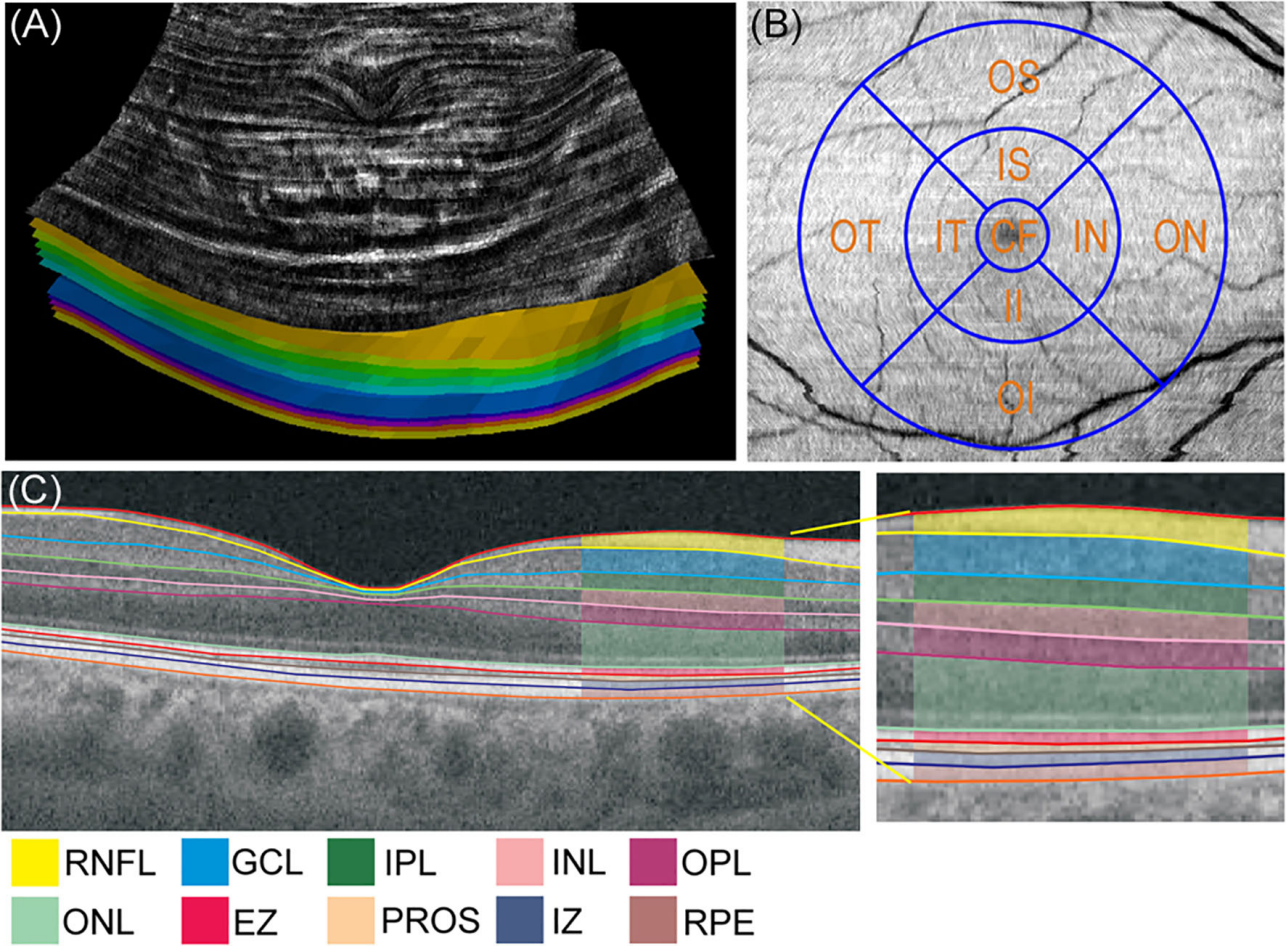
**(A)** Enface thickness image generated by OCT-Explorer of control subject. The innermost layer was inner limiting membrane, and the outermost layer was retinal pigment epithelium. **(B)** Three concentric circles of 1, 3, and 6 mm diameter and 9 Early Treatment Diabetic Retinopathy Study grids. **(C)** Representative optical coherence tomography (OCT) image presenting the mean thickness of macular neuroretinal layers and retinal pigment epithelium in control subject visualized by swept-source OCT and OCT-Explorer. CF, central fovea; EZ, ellipsoid zone; GCL, ganglion cell layer; II, inner inferior; IN, inner nasal; INL, inner nuclear layer; IPL, inner plexiform layer; IS, inner superior; IT, inner temporal; IZ, interdigitation zone; OI, outer inferior; ON, outer nasal; ONL, outer nuclear layer; OPL, outer plexiform layer; OS, outer superior; OT, outer temporal; PROS, photoreceptor outer segment; RNFL, retinal nerve fiber layer; RPE, retinal pigment epithelium.

The OCT images of the right eye were obtained every 3 months for 24 months using 3D swept-source-OCT (DRI OCT Triton, Topcon Corp., Tokyo, Japan) for all subjects. Macula was scanned using standard 6x6 mm protocol, in which 3D acquisition consisted of 256 B-scan slices. Only the high-quality images without artifact were included. The raw 256 jpg images were exported to OCT-Explorer (image size; 512 x 992 x 256 voxels) (10) which automatically assess the segmentation, enface thickness (**Figure 1A**), volume and reflectance images of the following MPRLs {1) ONL, 2) ellipsoid zone, 3) photoreceptor outer segment (PROS), 4) interdigitation zone} and RPE. The layer-specific mean reflectance was evaluated for each layer and each grid defined by the Early Treatment Diabetic Retinopathy Study (ETDRS). The raw scanned data were interpreted as 16-bit grayscale image, and reflectance was expressed in arbitrary unit (0〜256). These metrics were assessed at the central, pericentral, and peripheral rings with diameters of 1, 3, and 6 mm defined by the ETDRS at the following 9 grids: central fovea, inner and outer nasal, superior, temporal, and inferior grids (**Figure 1B**).

### Assessment of Neuropathy and Neurophysiological Examinations

The severity of the neuropathy and neurological deficits were assessed using the modified neuropathy disability score (11) which includes the evaluation of vibration, pin prick, temperature perception and ankle reflexes to establish the severity of neuropathy.

All subjects underwent neurophysiological examinations at the baseline and endpoint. Electrophysiology and nerve conduction velocity studies were performed using an electromyography instrument (Neuropak S1, NIHON KOHDEN, Tokyo, Japan). The motor (median nerve) and sensory (sural nerve) nerve conduction velocity, and their action potential amplitudes were determined. The patients with neuropathy disability score > 2 and sensory nerve conduction velocity of the sural nerve < 42 m/s were labeled with neuropathy based on the Toronto consensus (12) as previously reported (13).

The vibration perception threshold was measured at the left medial malleolus using a biothesiometer (Biomedical Instruments, Newbury, OH, USA). The warm and cold perception thresholds at the dorsum of the foot were determined using a thermal stimulator (Intercross-200, Intercross Co., Tokyo, Japan). The autonomic neuropathy and the cardiovagal function were assessed with the coefficient of variation of R-R intervals which was calculated from the R-R intervals of 200 samples on an electrocardiogram.

### Statistical Analysis

The *post hoc* analysis of sample power using G power 3.1 (http://gpower.software.informer.com/3.1/) revealed that the statistical power provided by the present study population was 0.96〜1.00 for the interaction of the metrics of MPRLs and RPE using the mixed analysis of variance (ANOVA) (significance of 0.05) and 0.83〜0.95 for the multiple regression analysis of the OCT metrics. All statistical analyses were performed using the SPSS (verion19, Chicago, IL, USA). All values are presented as the mean ± standard error of the mean (SEM). All data sets were tested for the normality using the Shapiro-Wilk test. For normally distributed variables, the comparisons between controls and patients with type 2 diabetes at the baseline or endpoint were made by Student t-test for continuous variables and the χ²-test for categorical variables. For non-normally-distributed continuous variables, Mann-Whitney’s U-test was applied and χ²-test for categorical variables. The differences between baseline and endpoint in each cohort were assessed using the paired t-test, and Wilcoxon signed-rank test for normally and non-normally distributed continuous variables, respectively, and χ²-test and McNemar test for normally and non-normally distributed categorical variables, respectively. The longitudinal changes in the metrics of MPRLs and RPE were estimated using a mixed ANOVA, which included 2 participant groups as a between-subjects factor, 9-time points as a within-subject factor and the OCT metrics as dependent variables. The sphericity of the within-subjects factors for 2 cohorts was estimated by Mauchly’s test. The degree of freedom was adjusted by Greenhouse-Geisser correction for violations of the sphericity assumption. The *post hoc* analyses were performed by Bonferroni’s multiple comparison test. The correlations between the changes in the OCT metrics at 9 ETDRS grids and parameters of clinical factors, HbA1c, CPPG, or neurological measures were assessed by the multiple regression analysis.

## Results

### Demographic Data

**Table 1** presents the summary of the demographic and clinical results. Patients with type 2 diabetes and control subjects were age- and gender-matched. At the baseline and endpoint the BMI, systolic blood pressure, CPPG, and HbA1c levels in patients were higher, and HDL-cholesterol was lower than controls. At the baseline LDL-cholesterol, triglycerides and urinary albumin-to-creatinine ratio in patients were higher than controls. During the follow-up period, systolic and diastolic blood pressure, CPPG, HbA1c levels, LDL-cholesterol, and estimated glomerular filtration rate in patients were significantly decreased. The insulin-sensitizing agent (6.5→58.1%) and dipeptidyl peptidase-4 inhibitor (DPP4-I, 9.7→80.6%) were more prescribed at the endpoint than baseline (**Table 1**).

**Table 1:** Demographic data and clinical characteristics at baseline and endpoint in patients with type 2 diabetes and control subjects.

	Patients with type 2 diabetes		Control subjects	
	Baseline	Endpoint	Baseline	Endpoint
Number (Male/Female, %)	31 (64.5/35.5)	31 (64.5/35.5)	39 (64.1 /35.9)	39 (64.1/35.9)
Age (year)	49.8±1.8	51.8±1.8	49.2±1.2	51.2±1.2
Body mass index (kg/m^2^)	25.6±0.88^*^	25.9±1.02^†^	22.5±0.41	22.6±0.43
Follow up period (year)	-	2.07±0.02	-	2.04±0.01
Duration of diabetes (year)	5.5±1.4	7.5±1.4	-	-
Systolic blood pressure (mmHg)	141±3.6^‡^	131±2.3^§, †^	120±1.7	122±1.5
Diastolic blood pressure (mmHg)	85.3±2.2^װ^	76.4±1.5^§^	78.5±1.0	79.5±0.9
No. treated with ARB (%)	5 (16.1)^װ^	7 (22.6)^¶^	0 (0)	1 (2.6)
Casual postprandial plasma glucose (mg/dL)	272±19.4^‡^	162±10.3^#,**^	97.5±2.3	104±2.5
Mean (mg/dL)		156±6.7		
Standard deviation (mg/dL)		43.8±3.2		
Coefficient of variation (%)		29.0±2.3		
HbA1c (%)	10.3±0.34^‡^	6.7±0.07^#,**^	5.4±0.05	5.5±0.03
Mean (%)	-	7.05±0.08	-	-
Standard deviation (%)	-	0.94±0.09	-	-
Coefficient of variation (%)	-	13.4±1.3	-	-
LDL-cholesterol (mmol/L)	3.46±0.16 ^װ^	3.08±0.16^††^	3.02±0.12	2.90±0.09
No. treated with statins (%)	2 (6.5)	1 (3.2)	3 (7.7)	3 (7.7)
HDL-cholesterol (mmol/L)	1.45±0.07 ^װ^	1.48±0.07^†^	1.71±0.07	1.74±0.06
Triglycerides (mmol/L)	2.25±0.34^*^	1.68±0.16	1.53±0.25	1.40±0.18
No. treated with fibrates (%)	0 (0)	0 (0)	0 (0)	0 (0)
Estimated glomerular filtration rate (mL/min)	91.5±4.17	85.1±3.16^††^	86.4±2.46	83.5±2.54
Urinary albumin to creatinine ratio (mg/gCr)	20.1±4.52^‡^	18.8±4.93^¶^	8.49±1.22	8.61±0.95
Hypoglycemic treatment None/SU/ISA/DPP4-I/diet alone (no.)	26/3/2/3/0	0^**^ /6/18^**^ /25^**^ /1

Data are the mean ± standard error of the mean in patients with type 2 diabetes and control subjects at baseline and endpoint.

^*^p < 0.01 compared with control subjects at baseline, ^†^p < 0.01 compared with control subjects at endpoint, ^‡^p <0.001 compared with control subjects at baseline, ^§^p < 0.01 compared with baseline, ^װ^p < 0.05 compared with control subjects at baseline, ^¶^p < 0.05 compared with control subjects at endpoint, ^#^p < 0.001 compared with control subjects at endpoint,^**^p < 0.001 compared with baseline, ^††^p < 0.05 compared with baseline.

ARB, angiotensin receptor blocker; DPP4-I, dipeptidyl peptidase-4 inhibitor; HDL, high-density lipoprotein; ISA, insulin-sensitizing agent; LDL, low-density lipoprotein; SU, sulfonylurea.

HbA1c levels at baseline (10.3 ± 0.34%) was intensively decreased to 7.0 ± 0.10% at 3 months with adequate speed (1.1%/month). Thereafter, HbA1c levels were kept between 6.5%〜6.7%.

### Neurophysiological and CNF Measures, and Microvascular Complications

Neuropathy disability score, neurophysiological and CNF measures at the baseline and endpoint in patients were altered compared with controls. Glycemic control did not alleviate neurophysiological measures, while improving some CNF measures. There was no diabetic retinopathy at the baseline and no new incidences of retinopathy in patients with type 2 diabetes. Glycemic control in patients with type 2 diabetes reduced the prevalence of neuropathy, but this did not reach the statistical significance (p = 0.453) (**Table 2**).

**Table 2:** Neurophysiological measures, corneal nerve fiber measures and microvascular complications at baseline and endpoint in patients with type 2 diabetes and control subjects.

	Patients with type 2 diabetes		Control subjects	
	Baseline	Endpoint	Baseline	Endpoint
**Neurophysiological measures**				
Neuropathy disability score	4.39±0.50^*^	3.84±0.42^†^	0.44±0.08	0.49±0.08
MCV of median nerve (m/s)	52.5±0.60^*^	53.7±0.54^†^	59.0±0.70	58.3±0.57
Amplitude of median nerve (mV)	8.03±0.57^‡^	7.12±0.60^†^	9.18±0.44	9.53±0.39
SCV of sural nerve (m/s)	45.1±1.00^§^	46.3±0.86^†^	49.4±0.72	50.0±0.63
Amplitude of sural nerve (μV)	11.5±0.93^§^	13.0±1.00	15.9±1.15	15.7±1.02
Vibration perception threshold (μ/120c/s)	3.30±0.37^*^	2.75±0.36	1.67±0.16	1.78±0.12
Coefficient of variation of R-R interval (%)	3.15±0.24^§^	3.06±0.25^†^	4.13±0.24	4.25±0.18
Warm perception threshold (W/m^2^)	-635±45.4^‡^	-616±42.3^װ^	-497±20.5	-489±16.0
Cold perception threshold (W/m^2^)	544±25.1	509±20.7	490±19.3	496±11.8
**Corneal nerve fiber measures**				
Corneal nerve fiber density (no/mm^2^)	19.9±0.53^*^	20.2±0.50^†^	30.8±0.83	30.3±0.57
Corneal nerve fiber length (mm/mm^2^)	10.7±0.26^*^	11.4±0.27^¶,†^	15.1±0.30	15.4±0.34
Corneal nerve branch density (no/mm^2^)	9.84±0.50^*^	11.3±0.42^¶,†^	14.2±0.77	14.5±0.56
Beading frequency (no/0.1mm)	19.4±0.25^*^	19.6±0.28^†^	23.6±0.33	24.0±0.27
Bead size (µm^2^)	11.7±0.10^*^	10.3±0.12^#,†^	8.09±0.074	8.11±0.051
**Microvascular complications**				
Prevalence of retinopathy (%)	0	0	0	0
Prevalence of neuropathy (%)	25.8	16.1	0	0
Prevalence of nephropathy (%)	16.1	19.4	0	0

Data are the mean ± standard error of the mean in patients with type 2 diabetes and control subjects at baseline and endpoint.

^*^p < 0.001 compared with control subjects at baseline, ^†^p < 0.001 compared with control subjects at endpoint, ^‡^p < 0.05 compared with control subjects at baseline, ^§^p < 0.01 compared with control subjects at baseline, ^װ^p < 0.05 compared with control subjects at endpoint, ^¶^p < 0.05 compared with baseline, ^#^p < 0.001 compared with baseline.

MCV, motor nerve conduction velocity; SCV, sensory nerve conduction velocity.

### The Mean Thickness at ETDRS Grids of MPRLs and RPE

**Figure 2:**
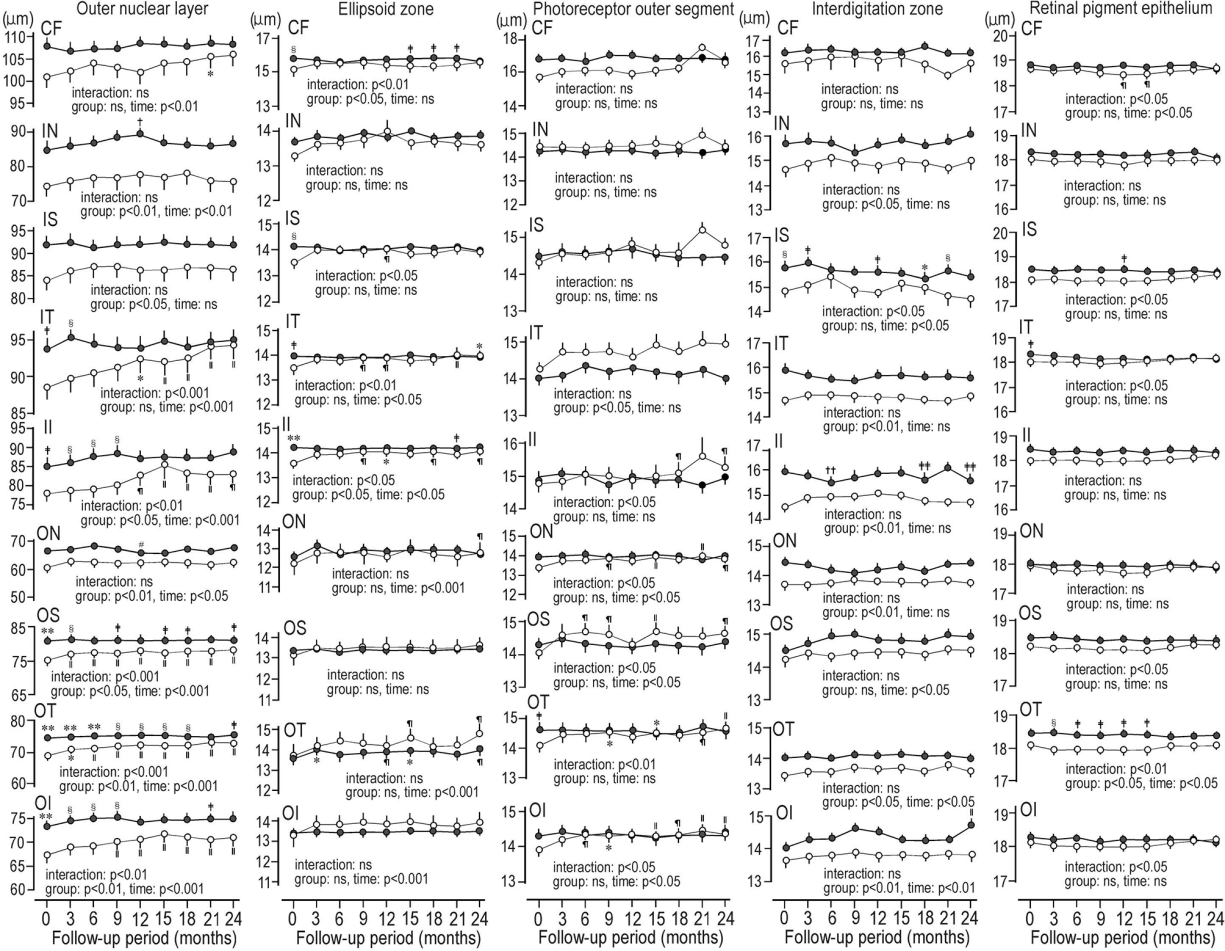
Sequential changes for 2 years in the mean thickness at grids defined by Early Treatment Diabetic Retinopathy Study of individual macular photoreceptor layers and retinal pigment epithelium in patients with type 2 diabetes undergoing glycemic control (open circle) and control subjects (solid circle). Values were mean ± SEM. ^*^p < 0.01 compared with baseline, ^†^p < 0.05 compared with 3 months, ^‡^p < 0.05 compared with patients with type 2 diabetes, ^§^p < 0.01 compared with patients with type 2 diabetes, ^װ^p < 0.001 compared with baseline, ^¶^p < 0.05 compared with baseline, ^#^p < 0.05 compared with 6 months, ^**^p < 0.001 compared with patients with type 2 diabetes, ^††^p < 0.01 compared with 21 months, ^‡‡^p < 0.05 compared with 21 months. CF, central fovea; II, inner inferior; IN, inner nasal; IS, inner superior; IT, inner temporal; OI, outer inferior; ON, outer nasal, OS; outer superior; OT, outer temporal.

**Figure 1C** shows the representative image of the mean thickness of neuroretinal layers and RPE in a control subject. **Figure 2** compared the sequential changes in the mean thickness at 9 ETDRS grids of MPRLs and RPE between control subjects and patients with type 2 diabetes undergoing glycemic control for 24 months. The thickness at 7 grids of ONL and at inner tempoal grid at the baseline and 3 months in patients was thinner than control. The thickness at 5 ONL grids in patients was significantly increased from the baseline. The baseline thickness at 4 grids of ellipsoid zone in patients was thinner than controls, and glycemic control restored them to the control levels at endpoint. Glycemic control increased the thickness at 5 PROS grids from the baseline. The thickness at 7 grids of interdigitation zone and one RPE grid in patients was thinner than controls. The glycemic control did not restore them (**Figure 2**).

As the volumes at grids of MPRLs and RPE were automatically determined by OCT-Explorer by multiplying the mean thickness by the area of each ETDRS grids, changes in the volume by glycemic control in patients were quite similar to those in the mean thickness.

### The Reflectance at ETDRS Grids of MPRLs and RPE

**Figure 3:**
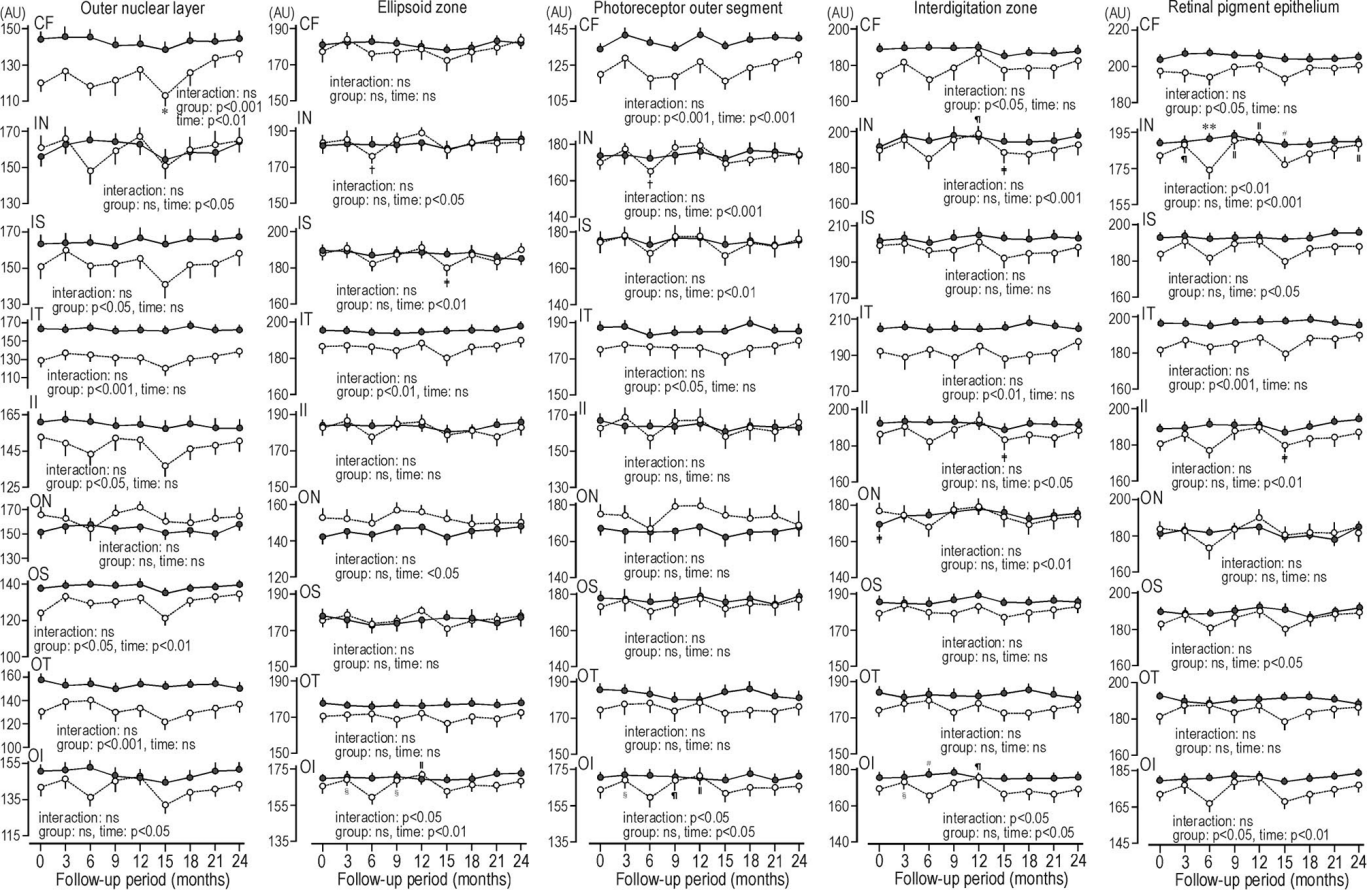
Sequential changes for 2 years in the reflectance at grids defined by Early Treatment Diabetic Retinopathy Study of individual macular photoreceptor layers and retinal pigment epithelium in patients with type 2 diabetes undergoing glycemic control (open circle) and control subjects (solid circle). Values were mean ± SEM. ^*^p < 0.05 compared with endpoint, ^†^p < 0.01 compared with 12 months, ^‡^p < 0.05 compared with 12 months, ^§^p < 0.05 compared with 6 months, ^װ^p < 0.001 compared with 6 months, ^¶^p < 0.01 compared with 6 months, ^#^p < 0.05 compared with patients with type 2 diabetes, ^**^p < 0.001 compared with patients with type 2 diabetes. AU, arbitrary unit; CF, central fovea; II, inner inferior; IN, inner nasal; IS, inner superior; IT, inner temporal; OI, outer inferior; ON, outer nasal, OS; outer superior; OT, outer temporal.

**Figure 4:**
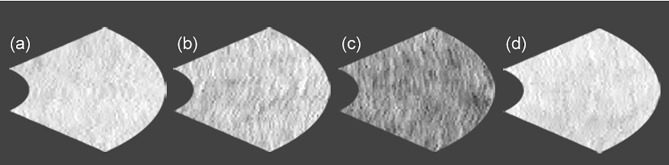
The comparison of the reflectances at inner nasal grid of retinal pigment epithelium between control subject and patient with type 2 diabetes under glycemic control. (a) control subject at 6 months (223), (b) patient with type 2 diabetes at 3 months (204), (c) patient with type 2 diabetes at 6 months (147), and (d) patient with type 2 diabetes at 9 months (224). No. in the parenthesis reveals reflectance in arbitrary unit.

In patients with type 2 diabetes, the reflectance at ONL grids at 6 or 15 months during glycemic control seems to decrease. The reflectances at 6 ONL grids in patients were weaker than controls. There was scarcely any group difference in the reflectances of ellipsoid zone. In ellipsoid zone of patients the reflectances at 3 grids were temporally decreased. In PROS of patients the reflectances at 2 grids were weaker than controls, and reflectances at 2 grids were temporally decreased. In patients, the reflectances at 2 grids of interdigitation zone were weaker than controls, and temporal decrease was found at one grid. At RPE of patients the reflectances at 3 grids were weaker than controls. The temporal decreases were seen at 2 grids. At inner nasal grid of parients the reflectances at 6 and 15 months were weaker than those of controls (**Figure 3**). At 6 months the refrectance at the inner nasal grid was clearly weaker than at 3 and 9 months in patients and at 6 months in controls (**Figure 4**).

### Multiple Regression Analysis between Metrics of MPRLs or RPE and Clinical Factors

By multiple regression analysis, the thickness at some ONL grids in patients increased negatively related with the decrease and mean of HbA1c levels over the whole follow-up period and those at 6-24 months, and positively related with baseline HbA1c levels. The mean CPPG and those at 12,18 and 21 months were negatively related with the increase in the thickness at the outer temporal ONL grid. The decrease in the reflectance at 6 months during glycemic control at outer inferior grid of ellipsoid zone or PROS was inversely associated with the HbA1c levels at that time (**Table 3**).

**Table 3:** Correlations between the increase in the thickness at grids of the outer nuclear layer or the decrease in reflectance at EZ and PROS grids and parameters of HbA1c, CPPG, and their variability during the follow-up period in patients with type 2 diabetes undergoing glycemic control for 24 months.

		Increase in the thickness at grids of outer nuclear layer by glycemic control				Decrease in reflectance at the outer inferior grid of EZ and PROS at 6 months during glycemic control			
		Inner temporal grid		Outer temporal grid		Outer inferior grid of EZ		Outer inferior grid of PROS	
		Standard β	p	Standard β	p	Standard β	p	Standard β	p
**HbA1c level**s	decrease	-0.545	**0.004**	-0.588	**0.004**	-0.041	0.854	-0.160	0.463
	mean	-0.473	**0.026**	-0.570	**0.008**	-0.021	0.929	0.101	0.667
	standard deviation	0.321	0.273	0.227	0.485	-0.478	0.126	-0.534	0.084
	coefficient of variation	0.486	0.077	0.447	0.138	-0.411	0.166	-0.501	0.087
	baseline	0.450	**0.020**	0.547	**0.008**	0.095	0.664	0.227	0.292
	3 months	-0.102	0.648	-0.153	0.526	-0.226	0.338	0.011	0.964
	6 months	-0.388	0.082	-0.631	**0.006**	-0.803	**<0.001**	-0.665	**0.001**
	9 months	-0.581	**0.033**	-0.634	**0.003**	-0.044	0.847	0.099	0.661
	12 months	-0.534	**0.007**	-0.639	**0.003**	0.072	0.754	0.163	0.472
	15 months	-0.572	**0.004**	0.816	**<0.001**	-0.021	0.929	0.214	0.346
	18 months	-0.578	**0.002**	-0.686	**0.001**	-0.013	0.952	0.101	0.647
	21 months	-0.561	**0.003**	-0.656	**0.001**	-0.024	0.913	0.226	0.303
	endpoint	-0.705	**<0.001**	-0.783	**<0.001**	0.264	0.267	0.279	0.237
**CPPG**	decrease	-0.171	0.394	-0.328	0.124	-0.257	0.225	-0.331	0.111
	mean	-0.070	0.736	-0.498	**0.019**	-0.145	0.509	-0.138	0.526
	standard deviation	0.138	0.512	0.076	0.740	-0.295	0.180	-0.117	0.599
	coefficient of variation	0.223	0.276	0.255	0.250	-0.279	0.198	-0.105	0.631
	baseline	0.109	0.573	0.170	0.416	0.182	0.376	0.234	0.249
	3 months	0.080	0.688	-0.227	0.289	-0.195	0.354	-0.312	0.131
	6 months	-0.033	0.874	-0.398	0.070	0.255	0.221	0.231	0.191
	9 months	0.003	0.987	-0.321	0.125	-0.132	0.531	-0.196	0.347
	12 months	-0.251	0.263	-0.464	**0.049**	-0.220	0.358	-0.364	0.120
	15 months	0.030	0.887	-0.215	0.343	-0.323	0.142	-0.257	0.243
	18 months	-0.202	0.335	-0.548	**0.010**	-0.132	0.555	0.164	0.460
	21 months	-0.153	0.463	-0.494	**0.021**	-0.067	0.763	-0.071	0.748
	endpoint	-0.174	0.443	-0.420	0.078	-0.222	0.354	-0.288	0.225

Decrease in HbA1c = value at endpoint – value at baseline. Increase in thickness = value at endpoint – value at baseline. Decrease in reflectance = reflectance at 6 months – reflectance at 3 months after the start of glycemic control. CPPG, casual postprandial plasma glucose; EZ, ellipsoid zone; PROS; photoreceptor outer segment.

Statistically significant correlations appear in boldface type.

The mean levels (β:-0.220〜0.060, p:0.268〜0.782) or improvements (β:-0.257〜0.092, p:0.232〜0.784) of the metabolic syndrome components during glycemic control did not influence the ONL thickness and the reflectances of ellipsoid zone or PROS. The interval changes in neurophysiological tests (β:-0.227〜0.348, p:0.133〜0.942) and CNF measures (β:-0.349〜0.275, p:0.090〜0.992) were not associated with the increase in thickness at inner temporal and outer temporal grids of ONL nor weaker reflectance at outer inferior grid of ellipsoid zone or PROS. The increased prescription of DPP4-I and insulin sensitizing agent at the endpoint did not influence the ONL thickness (β:-0.353〜0.339, p:0.067〜0.603) nor the reflectance of ellipsoid zone or PROS (β:-0.249〜0.197, p:0.400〜0.930).

## Discussion

The present study investigated the sequential changes during glycemic control in the mean thickness, volume and reflectance of individual MPRLs and RPE using enface OCT in type 2 diabetic patients without diabetic retinopathy. In patients with diabetes, evidences showing photoreceptor death is equivocal (4, 14). Therefore, not much has been clarified what occurs in the metrics of individual MPRLs and RPE during glycemic control in type 2 diabetes. Indeed the glycemic control has the potential transient negative influence on diabetic retinopathy (15). In patients with type 2 diabetes 4 months after starting glycemic control, retinal nerve fiber layer got thinner related with the decrease in HbA1c levels (16). Therefore, the sequential OCT examinations during glycemic control is crucial to clarify the dynamic influence of glycemic control on the metrics and neuroretinal structures. This may enable to predict the future incidences of diabetic retinopathy. Because the thickness of most MPRLs and RPE is < 20 µm, the precise measurement and good repeatability are necessary to elucidate the impact of glycemic control on the small but significant changes in the OCT metrics in diabetic patients. In control subjects, the coefficients of variation in 9 measurements of the mean thickness and volume over 2 years at 9 ETDRS grids of ONL, ellipsoid zone, PROS, interdigitation zone and RPE were 1.6-5.3%, 1.6-3.7%, 1.9-3.8%, 1.7-5.5%, and 1.2-1.8%, respectively, indicating good repeatablility, although some significant fluctuations even in control subjects were seen. In contrast, the coefficients of variation in 9 reflectance measurements at these layers in controls were 8.8-11.0%, 4.5-6.5%, 4.8-7.3%, 4.4-5.8% and 4.6-6.0%, respectively, indicating poorer repeatablility than the mean thickness and volume. The sequential changes in the reflectance by the intervention such as glycemic control should be cautiously interpreted.

In the present study, the thickness of 7 grids of ONL over whole follow-up period, and partially at inner temporal grid in patients was thinner than controls. Glycemic control significantly increased the thickness at 6 grids of ONL from the baseline. In patients the mean thickness at the inner temporal grid was restored to the control level after 24 months follow-up. Because glycemic variability plays a causative role in diabetic complications independent of HbA1c levels (17), we associated the recovery of some ONL thickness with parameters of glycemic control and glycemic variability. The recovery of some ONL grid thickness was robustly associated with overall HbA1c decrease from the baseline and monthly low HbA1c levels during the second year of follow-up period. The mean HbA1c and CPPG levels also contributed to ONL recovery. The baseline HbA1c levels did not influence any baseline ONL thickness (P = 0.205〜0.966), but positively related with the recovery of some ONL thickness. Because the HbA1c levels at the endpoint in patients were similar (6.5〜6.7%), the decrease in HbA1c levels was highly correlated with the baseline HbA1c levels (p < 0.001). Then, the high baseline HbA1c levels positively correlated with the increase in ONL thickness. In contract, glycemic variability was not significantly related with the recovery of ONL thickness. However, glycated albumin has been reported to be better marker of glycemic variability than HbA1c levels, especially for short term (18). Because the present study did not measure glycated albumin, we could not clarify the relationship between the variability of glycated albumin and the metrics of MPRLs and RPE.

A reduced ONL thickness may represent photoreceptor cell death (19). Therefore, the ONL thickness, especially at inner temporal grid, can provide a measure for the photoreceptor recovery by glycemic control. The cone density near central fovea is highest and abruptly decreases toward the periphery, and the rod occupies 90% of photoreceptors (20). Because glycemic control increased the thickness at central fovea, pericentral and peripheral grids, glycemic control might restore ONL of the cones and rods.

The thickness at 4 grids of ellipsoid zone at the baseline in patients with type 2 diabetes was thinner than control subjects. Glycemic control significantly increased the thickness at 3 inner and 2 outer grids of ellipsoid zone from the baseline. The ellipsoid zone contains mitochondria which are the main focus of hyperglycemia-induced retinal oxidative stress (21). The recovery of ellipsoid zone by glycemic control may represent the amelioration in photoreceptor oxidative stress and mitochondrial degeneration.

As previously reported (3, 4), the most baseline PROS thickness in patients with type 2 diabetes was similar to that of control subjects, and glycemic control increased the thickness from the baseline at all peripheral and one pericentral grids, suggesting the PROS of the rod in patients might be more amenable to glycemic control than cone which located near the central fovea. This was compatible with less severe demand in blood supply and metabolism in the peripheral retina than central fovea (4).

The mean thickness of 6 grids of interdigitation zone and one grid of RPE in patients was thinner than controls (22). Glycemic control did not restore the thickness of interdigitation zone and RPE. The influence of glycemic control on these layers had never been investigated and the current study is the first to our knowledge that reports these findings.

The influence of glycemic control on the volume of MPRLs and RPE was quite similar to that on the mean thickness, because OCT-Explorer automatically calculated the volume by multiplying the mean thickness by the each grid area defined by the ETDRS.

The second aspect of these novel findings is the sequential assessment of reflectance in MPRLs and RPE during glycemic control in diabetic patients without diabetic retinopathy. This may disclose the alterations occurring before the development of new diabetic retinopathy, as reported in cross-sectional study of type 1 diabetes (23). The reflectance at baseline and during follow-up in patients was similar or less than controls. The transient weaker reflectance occurred at 6 and 15 months during glycemic control. To rule out the shadowing effect by high reflectance at inner retinal layers to outer retinal layers (24), we measured reflectances at all inner retinal layers. There were no high reflectances, and weaker reflectances were found at all inner retinal layers at the same time points as MPRLs and RPE. The weaker reflectance at outer inner grid of ellipsoid zone and PROS at 6 months during glycemic control was robustly related with high HbA1c levels at that time point. This means that the temporal glycemic deterioration during glycemic control may cause weaker reflectance of MPRLs, suggesting the possible derangement of MPRLs. The erratically high blood glucose rather than constant high glucose exposure was shown to have more harmful consequences associated with oxidative stress and endothelial dysfunction (25). Because the ellipsoid zone contains mitochondria which are the main focus of hyperglycemia-induced retinal oxidative stress (21), the temporal glycemic deterioration during glycemic control may result in the derangement of MPRLs. In the perifoveal region, the reflectances in ellipsoid zone and interdigitation zone are closely related to the cone density (26). The weaker reflectance may result from the disarray of the mosaic cone and rod distribution, as found in various retinal diseases (27). If the hyperglycemia-induced hyperosmolarity may enhance dehydration in situ, the baseline thickness was reduced, and reflectance was increased. The subsequent glycemic control may restore normal osmolarity and improve the thickness and reflectance toward the endpoint. We think this was unlikely, because the thickness in some grids at baseline was sequentially and significantly increased toward the endpoint, while the reflectance at endpoint did not significantly changed from baseline at all grids of MPRLs and RPE, and the weaker reflectances were transient at specific time points and reversible, and because chronic hyperglycemia could increase the permeability of retinal vasculature (28), which may have led to the thickening of the retinal layers due to edema (3) at baseline.

The thickness and reflectance in patients with diabetes seem to be variable compared with those in control subjects. The metrics of MPRLs and RPE are influenced by not only glycemic levels (28), but also many clinical factors including anthropometric values, blood pressure and serum lipid levels (22). In control subjects, these clinical factors were stable and existed in a narrow range during the follow-up period, while in the present study all values in patients except for BMI were significantly different between baseline and endpoint. Furthermore, clinical background of diabetes such as disease duration and severity of hyperglycemia was different among patients with diabetes. These diverse clinical factors in patients may induce variability in the metrics of MPRLs and RPE. The fovea has the highest density of cones, and therefore has an increased metabolic demand, and the temporal regions have the thinnest retina. The foveal and temporal regions of retina may be susceptible to diabetic harm (28). Therefore, the influence of diabetes on neuroretina may be variable among ETDRS grids, and cause regional variability in the metrics of MPRLs and RPE.

The causative mechanisms of the transient weaker reflectances of MPRLs and RPE are unknown. According to the neurodegenerative theory of diabetic retinopathy, the apoptosis of neuronal cells, including photoreceptor cells, observed at an early stage of diabetes, might lead to microvascular changes (29). In type 1 diabetes without diabetic retinopathy, the weaker reflectance in some retinal layers is supposed to predict the new development of diabetic retinopathy (23). Therefore, transient weaker reflectances in the present study may be relevant to the development of diabetic retinopathy induced by glycemic control.

Because the increase in ONL thickness during glycemic control was not associated with the improvement in CNF and neurophysiological measures, and because glycemic control did not ameliorate the neurophysiological measures, the recovery of ONL thickness by glycemic control might be a novel morphological marker for investigating the benefit of glycemic control on the diabetic neuropathy. Although glycemic control improved some CNF measures, all measures at endpoint were still inferior to those in control subjects. In contrast, glycemic control increased the mean thickness and volume at some grids of ONL, ellipsoid zone and PROS to the levels of control subjects, indicating that glycemic control had potential to normalize the thickness and volume of some MPRLs.

Because the normal HbA1c levels (< 5.9%) (13) or HbA1c levels < 6.5% (30) are mandatory for improving neurophysiological and CNF measures by glycemic control, the average HbA1c levels in the current study beyond these levels could not improve the neuropathy measures.

Although chronic hyperglycemia clearly plays a causative role in the retinal neurodegeneration in type 2 diabetes, metabolic syndrome components are likely to cause it (31). In our study, the mean levels or changes in metabolic syndrome components during follow-up period were not associated with the increase in ONL thickness.

### Strengths and Limitations

The longitudinal impact of glycemic control on the OCT metrics in type 2 diabetes had never been investigated. For excluding time-dependent variability in OCT metrics, this study repeated OCT examinations every 3 months for 2 years. The current study measured the main factors influencing the metrics of MPRLs and RPE monthly or every 3 months during the follow-up period. Therefore, the mean clinical factors influencing the OCT metrics are representative; the contribution of controlling hyperglycemia and other risk factors to the restoration of altered MPRLs and RPE was reliably evaluated by multiple regression analysis.

Our study has some limitations. First, the follow-up period of 2 years may need to be extended. Ideally, the glycemic control needs to be maintained for over 3-5 years to yield definite benefit (32). Two years might be too short to restore the morphology of all MPRLs and RPE, and neurological deficits. Second, we did not examine the retinal function such as electroretinography. The simultaneous examinations using electroretinography and OCT strengthen the role of glycemic control on the restoration of MPRLs and RPE. Third, because we aimed to unveil changes in MPRL metrics before the development of diabetic retinopathy, patients with diabetic retinopathy were excluded. Therefore, we could not assess the influence of diabetic retinopathy on the metrics of MPRLs. Fourth, the weaker reflectances at the specific time points occurred in relation to coincident HbA1c increase during glycemic control. The future research, including many diabetic patients developing new diabetic retinopathy during glycemic control is indicated to clarify the role of transient weaker reflectance for the development of diabetic retinopathy induced by glycemic control.

In conclusion, the longitudinal impact of glycemic control on the OCT metrics in type 2 diabetes had never been investigated. In this paper for the first time, we conducted such a follow-up study. The glycemic control in type 2 diabetes sequentially restored the mean thickness and volume at some MPRL gids over 24 months follow-up period. Some reflectances of MPRLs and RPE at specific time points during glycemic control were weaker than those at adjacent time points coincident with high HbA1c levels. The causative mechanisms of transient weaker reflectance during glycemic control should be investigated for predicting the development of diabetic retinopathy.

## Data Availability Statement

The raw data supporting the conclusions of this article will be made available by the authors, without undue reservation.

## Ethics Statement

Written informed consent was obtained from all subjects based on the Declaration of Helsinki. The ethics committee of Ishibashi Clinic approved the protocol of this study.

## Author Contributions

FI designed the study, researched data, and wrote the entire manuscript. AK gathered clinical and laboratory data and statistically analyzed all data. MT advised on the statistical analysis, interpreted the results, and reviewed and revised the whole manuscript. FI and MT are the guarantors of this work, and, as such, had full access to all data in the study and take responsibility for the integrity of the data and the accuracy of the data analysis and interpretation. All authors contributed to the article and approved the submitted version.

## Conflict of Interest

The authors declare that the research was conducted in the absence of any commercial or financial relationships that could be construed as a potential conflict of interest.

## Abbreviations

ANOVA, analysis of variance; CNF, corneal nerve fiber; CPPG, casual postprandial plasma glucose; DPP4-I, dipeptidyl peptidase4-inhibitor; ETDRS, Early Treatment Diabetic Retinopathy Study; MPRL, macular photoreceptor layer; OCT, optical coherence tomography; ONL, outer nuclear layer; PROS, photoreceptor outer segment; RPE, retinal pigment epithelium.
